# A novel approach to assess cerebral and coronary perfusion after cardiac arrest

**DOI:** 10.1186/s40635-018-0204-3

**Published:** 2018-10-12

**Authors:** Julien Adjedj, Fabien Picard, Maarten Vanhaverbeke, Bernard De Bruyne, Alain Cariou, Ming Wu, Stefan Janssens, Olivier Varenne

**Affiliations:** 1 AP-HP, Service de Cardiologie, Hôpital Cochin, Paris, France; 2 Faculté de Médecine Paris Descartes, Université Paris Descartes, Paris, France; 3 Department of Cardiovascular Sciences, KU Leuven, Leuven, Belgium; 4 OLV Cardiovascular Center, Aalst, Belgium; 5 AP-HP, Service de réanimation médicale, Hospital Cochin, Paris, France

**Keywords:** Cardiac arrest, Animal model, Absolute coronary flow, Absolute cerebral flow

## Abstract

**Background:**

Several indices exist to assess cerebral perfusion after cardiac arrest (CA). We aimed to investigate a new approach allowing absolute flow and microvascular resistance measurement based on selective arterial continuous thermodilution before and after CA resuscitation in a porcine model.

**Methods:**

In anaesthetised pigs, intravascular absolute cerebral blood flow (CBF) and absolute coronary blood flow (ABF) with corresponding microvascular resistances were measured. CA was induced using overdrive pacing with 3 (group 1, *n* = 5) or 5 min (group 2, *n* = 8) of no flow. After resuscitation, CBF was performed at baseline, at 15 min (T15) and at 30 min (T30). Thereafter, CBF in the contralateral cerebral artery and ABF were measured.

**Results:**

The protocol could not be completed in three pigs from group 2 due to haemodynamic instability. In the entire cohort, CBF was significantly lower at T30 after CA (0.026 ± 0.02 L/min vs 0.040 ± 0.03 at baseline; *p* = 0.03) and cerebral microvascular resistances were significantly higher (3202 ± 1838 Woods units vs 2014 ± 1015 at baseline; *p* = 0.04). ABF and resistances remained stable at baseline, as compared to T30 (0.122 ± 0.05 vs. 0.143 ± 0.06 L/min; *p* = 0.15 and 563 ± 203 vs. 478 ± 181 Woods units; *p* = 0.36, respectively). At T30, no significant differences in cerebral flow dynamics were observed between groups.

**Conclusions:**

ABF and CBF measurement after CA resuscitation is feasible with thermodilution technique, allowing accurate monitoring and measurements. This novel approach allows simultaneous measurements of flow and microvascular resistances. This animal model simplifies cerebral perfusion measurements and allows to test new therapies to reduce cerebral injury post cardiac arrest.

**Electronic supplementary material:**

The online version of this article (10.1186/s40635-018-0204-3) contains supplementary material, which is available to authorized users.

## Background

Despite advances to improve cardiovascular mortality and neurological outcome, out of hospital cardiac arrest (OHCA) management remains challenging. Short-term prognosis of patients who experienced OHCA remains poor, with more than 50% mortality [1]. While long-term prognosis after initial survival is preserved, the majority of OHCA survivors still do not fully recover their cognitive function [2]. Cerebral blood flow (CBF) is regulated to support the delivery of oxygen, washout of carbon dioxide and transport of vasoactive substances through metabolic, myogenic and neurogenic control of vascular resistances. The capacity of autoregulation to maintain cerebral perfusion is essential to sustain brain function. The brain is the first organ that suffers after OHCA, as it is particularly sensitive to hypoxia and ischemia which induces oedema and injury [3, 4]. Thus, no flow and slow flow duration are essentials for neurologic prognosis. Acute cerebral injury associated with OHCA typically results in a narrowing of the autoregulatory pressure range [5, 6]. As a consequence, immediate cerebral hyperaemia after cardiac arrest is typically followed by hypoperfusion, and finally, flow increases several hours later [7, 8]. An objective evaluation of acute cerebral perfusion appears to be essential to assess neurological prognosis, and guides clinical research and therapy. We sought to evaluate the measurement of CBF and microvascular resistances after cardiac arrest resuscitation in an animal model. Recently, a monorail infusion catheter (RayFlow, Hexacath, Paris, France) was developed to assess absolute coronary blood flow (ABF) and myocardial resistances based on thermodilution technique. ABF and microvascular resistance measurements are accurate in vitro and in vivo and can be performed safely in humans [9, 10]. We aimed to apply the same protocol in cerebral arteries to measure and monitor CBF and cerebral microvascular resistances in an animal model before and after resuscitated cardiac arrest. We hypothesised that CBF would decrease and that microvascular cerebral resistances would increase early after cardiac arrest while coronary flow will remain stable. These absolute values might also be of interest in an animal setting to evaluate cerebral perfusion and better understand haemodynamic changes after cardiac arrest resuscitation.

## Methods

### Cerebral anatomy

There are marked differences between pig and human cerebral vascular cerebral anatomies. In pigs, the common carotid branches into the external carotid artery and the ascending pharyngeal artery. The arteria anastomica arises from the ascending pharyngeal artery and is easily recognised with angiography (Fig. 2). The left and right arteria anastomica are upstream to the overall intracerebral arteries (including the internal carotid artery). We called the left arteria anastomica “left intracerebral artery” (LIC), and the right arteria anastomica “right intracerebral artery” (RIC) for clarification [11].

### Animal setting

Thirteen crossbred domestic pigs (*Sus scrofa*, weight 30–40 kg, Animalium KU Leuven, Leuven, Belgium) were sedated with Telazol (tiletamine 4 mg/kg and zolazepam 4 mg/kg; Zoletil100, Virbac Animal Health, Carros, France) and xylazine (2.5 mg/kg; Vexylan, CEVA Sante Animale, Brussels, Belgium) and anaesthetised with intravenous propofol bolus (3 mg/kg; Diprivan, AstraZeneca, Brussels, Belgium) followed by 10 mg/kg/h continuous infusion and remifentanil (18 μg/kg/h) (Ultiva, GSK, Genval, Belgium). Mechanical ventilation with a mixture of air and oxygen (1:1) at a tidal volume of 8–10 ml/kg was adjusted to maintain normocapnia and normoxia. Heart rate (HR) and cardiac rhythm were continuously monitored using electrocardiography. Anticoagulation (heparin 10,000 IU) and antiplatelet therapy (acetylsalicylic acid 500 mg) were administered at the beginning of the procedure intravenously.

### Protocol

Seven French (Fr) sheaths were introduced into the femoral vein and artery in each pig. A pacing probe was advanced under fluoroscopy in the right ventricle through the venous sheath. A Judkins 4 right, 6F guiding catheter (Cordis, Fremont, CA, USA) was advanced into the common carotid arteries left and right. Coronary and cerebral angiography were systematically performed following intra-arterial bolus administration of nitrates, and repeated in case of spasm.

#### Cerebral flow measurement

A 0.35-mm-diameter pressure wire (PressureWire X, Abbott, IL, USA) was introduced into the guiding catheter and advanced successively at the level of the arteria anastomica upstream to the right (RIC) and left intracerebral arteries (LIC). The 0.84-mm-diameter monorail infusion catheter was connected to an infusion pump loaded with isotonic saline at room temperature. CBF and microvascular resistances were measured in the ascending pharyngeal arteries with the pressure wire placed close to the arteria anastomica.

#### Coronary flow measurement

The same pressure wire over the monorail catheter was advanced in the left anterior descending or right coronary artery to measure ABF and microvascular resistances in the coronary arteries at baseline and after cardiac arrest resuscitation and measurements of CBF and microvascular resistances.

**Fig. 1:**
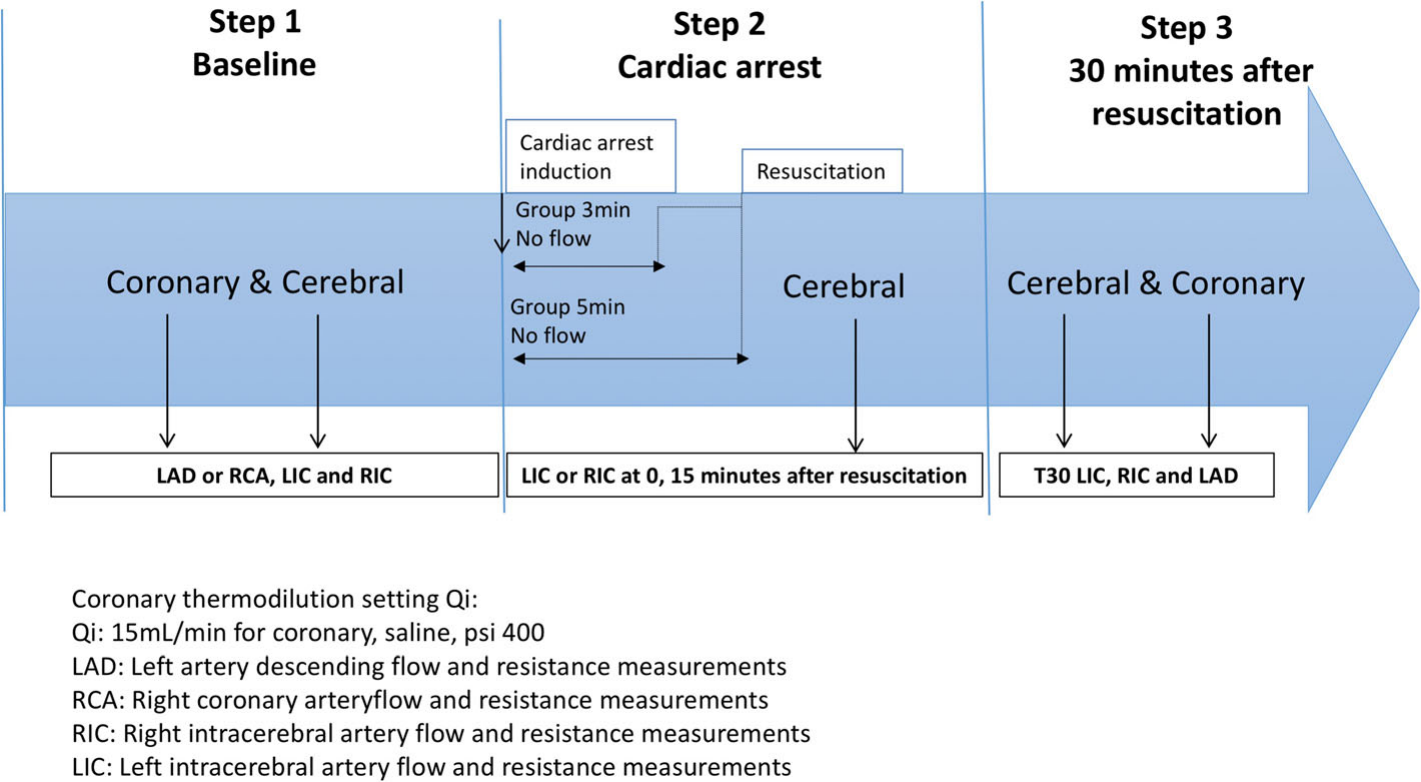
Study protocol

All pressure and temperature measurements were recorded after zeroing the temperature and equalising pressures using a Radi-Analyzer console (Abbott, IL, USA). We infused saline with an infusion pump (Medrad Stellant; Medrad Inc., Warrendale, PA, USA) at constant flow (15 mL/min, psi 600) for coronary measurements [12]. Flow was expressed in litres per minute and microvascular resistances in mmHg·min/L or Woods units (WU). Ventricular fibrillation was induced by administration of a pulse of 30 V at 200 Hz for 1 s in the right ventricle. Thereafter, according to the dedicated group, pigs were resuscitated after 3 or 5 min with standard resuscitation techniques, including the use of asynchronous defibrillation and adrenaline. Two groups were defined according to the no flow duration. Group 1 had 3 min of no flow, and group 2 had 5 min of no flow. CBF and resistances were measured in the RIC or the LIC at 0, 15 and 30 min after resuscitation. Thereafter, contralateral cerebral artery CBF and resistances were measured, and finally, ABF and resistances were measured. Final cerebral and coronary angiograms were systematically performed after each measurement (Fig. 1). At the end of the procedure, the pig was sacrificed by the injection of 10 mL of oversaturated KCL. All animal procedures were conformed to the guidelines from Directive 2010/63/EU of the European Parliament on the protection of animals used for scientific purposes or the NIH guidelines. The animal study was approved by the Animal Ethics Committee of the KU Leuven.

### Absolute flow and microvascular resistances

To measure flow and resistances, we placed the pressure wire in the distal part of the interrogated vessel of interest. The infusion catheter was advanced over the pressure wire to continuously infuse room temperature saline at a fixed rate (*Q*_i_), using an infusion pump. Temperature and distal pressure (*P*_d_) were simultaneously measured at the distal part of the pressure wire by a pressure/temperature sensor. During infusion, the temperature of the mixture of saline and blood was measured at steady state (*T*). Thereafter, the pressure wire was pulled back into the infusion catheter during infusion to measure the temperature of saline at the exit level of the infusion catheter (*T*_i_). Flow (*Q*) was calculated using the following formula:

$$ Q=1.08\frac{T_{\mathrm{i}}}{T}{Q}_{\mathrm{i}}. $$

Resistances were calculated using the following formula:

$$ R=\frac{P_{\mathrm{d}}}{Q} $$

**Fig. 2:**
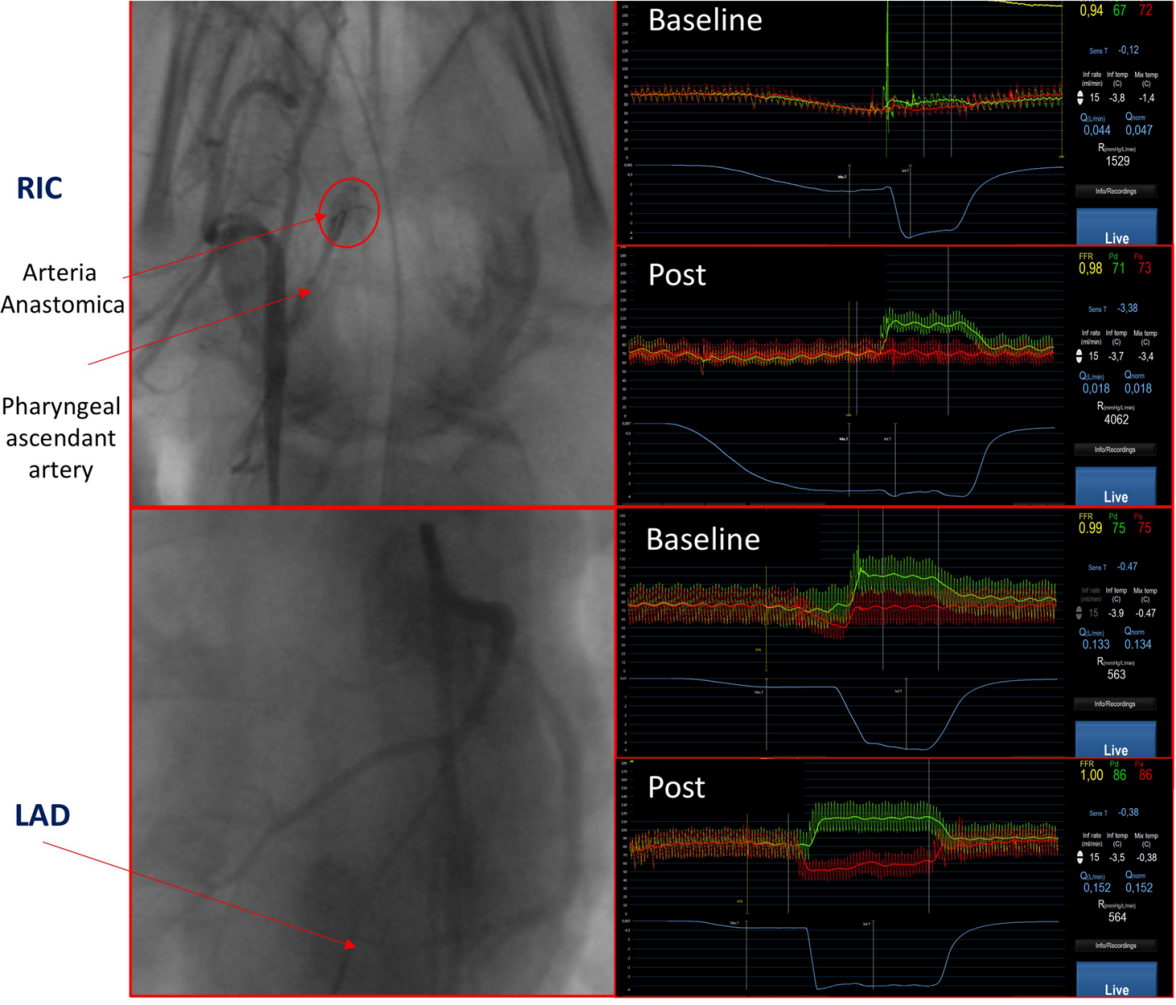
Illustration of absolute cerebral flow and microvascular resistance measurement at baseline and post cardiac arrest resuscitation (upper panel). Absolute coronary flow and microvascular resistances at baseline and post cardiac arrest resuscitation (lower panel). Cerebral angiography showing the arteria anastomica from the right internal carotid (upper panel left side) with the presence of a pressure/temperature wire and an infusion microcatheter. Coronary angiography showing the left descending artery (lower panel left side) with the presence of a pressure/temperature wire and an infusion microcatheter. The right side shows thermodilution tracings at baseline and post cardiac arrest resuscitation with arterial central pressure in red, arterial distal pressure in green and temperature curve in blue. Values of flow and resistances are express in litres per minute and Woods units respectively

All thermodilution tracings and angiographic images were analysed after the entire protocol. We used COROFLOW Software (Coroventis, Uppsala, Sweden) to calculate ABF and coronary microvascular resistances as well as CBF and cerebral microvascular resistances (Fig. 2).

### Statistical analysis

All values are expressed as mean ± standard deviation, as median value (continuous values) or as counts and percentages (categorical variables). Continuous variables were compared by the use of the nonparametric Mann-Whitney test or *t* test when appropriate. A *p* value < 0.05 was considered statistically significant. All statistical analyses were performed using SAS software version 7.1 and GraphPad Prism version 7.0.

## Results

We performed the protocol in 13 juvenile pigs of 34 ± 5 kg. Five pigs were included in group 1, and eight pigs were included in group 2. In three pigs from group 2, we could not finalise the protocol for the following reasons: one had refractory cardiogenic shock after cardiac arrest resuscitation, one could not be resuscitated due to electrical storm and the last one had persistent asystole after 45 min of cardiopulmonary resuscitation. Therefore, we excluded these three pigs from further analysis to finally obtain two groups of 5 pigs (Table 1). All pigs included in the analysis were successfully cardioverted with an external electrical shock at 360 J. All pigs in group 1 had one external electrical shock administrated for defibrillation; one pig received an additional 1 mg intravenous bolus injection of adrenaline. In group 2, several external electrical chocks were administrated, (on average 2.4 per pig), and all pigs received an intravenous bolus injection of adrenaline (on average 3.4 mg per pig). As a result, the time to return to spontaneous circulation was as follows: mean no flow duration of 3 min and 10 s ± 15 s (group 1), and mean no flow duration of 8 min and 36 s ± 180 s (group 2). After resuscitation, no vasoactive drug was administrated in the included pigs.

**Table 1:** Absolute cerebral flow and microvascular resistances at baseline and after cardiac arrest resuscitation at T0, T15 and T30 minutes

	n vessels	Baseline	n vessels	T0	n vessels	T15	n vessels	T30
Flow (L/min)								
overall	20	0.040 ± 0.03	6	0.090 ± 0.06	10	0.059 ± 0.05	19	0.026 ± 0.02
3	10	0.034 ± 0.02	3	0.096 ± 0.09	5	0.022 ± 0.01	9	0.028 ± 0.02
5	10	0.047 ± 0.03	3	0.083 ± 0.01	5	0.095 ± 0.06	10	0.025 ± 0.01
Resistances (mmHg·min/l or WU)								
overall	20	2014 ± 1015	6	1991 ± 1191	10	1967 ± 1048	19	3202 ± 1838
3	10	1771 ± 910	3	2256 ± 1671	5	2564 ± 940	9	2850 ± 1456
5	10	2278 ± 1561	3	1726 ± 737	5	1370 ± 832	10	3658 ± 2218

### Cerebral blood flow

**Fig. 3:**
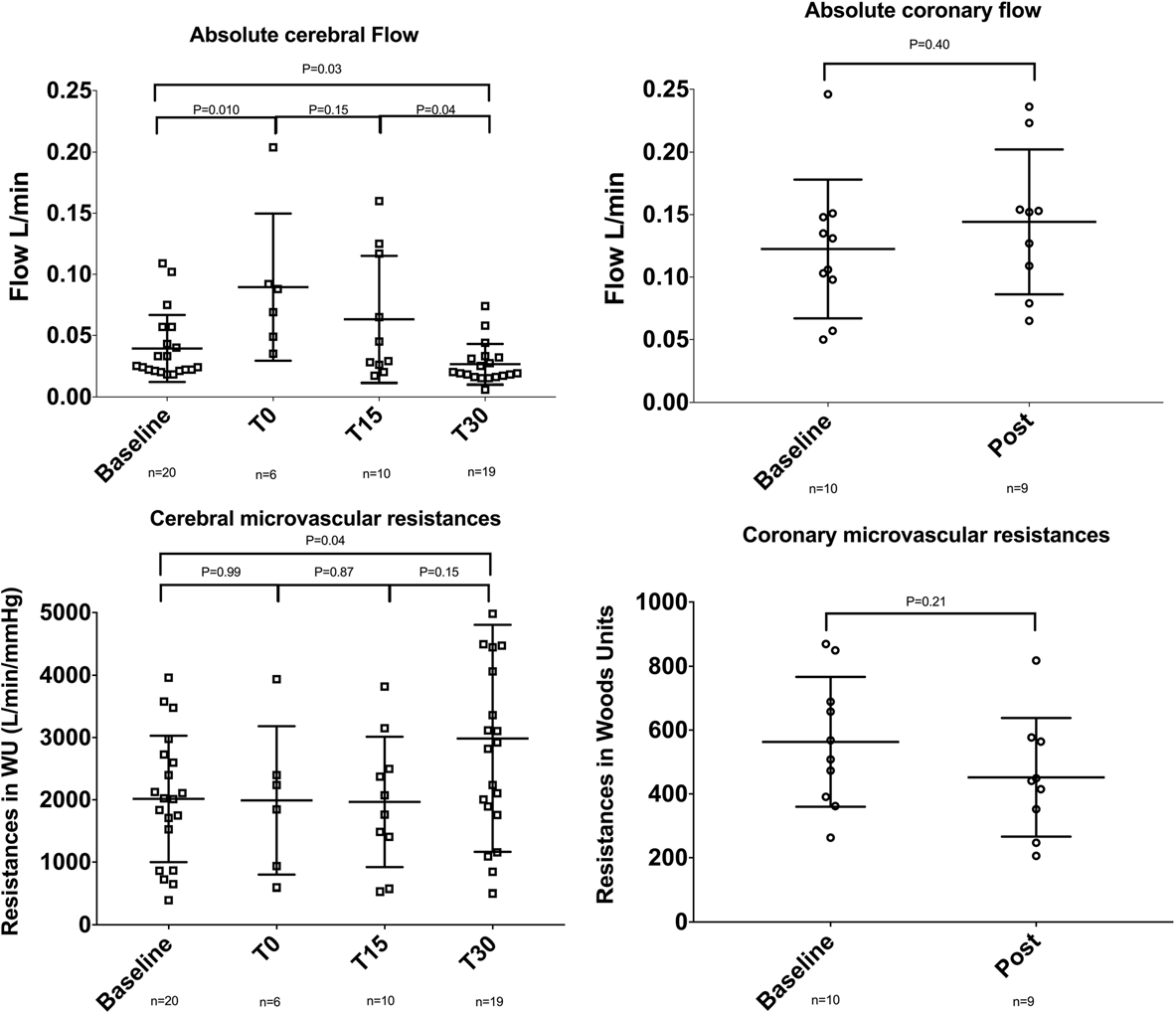
Flow modifications after cardiac arrest in all animals. The upper left panel shows the absolute cerebral flow modification at baseline and after cardiac arrest ressucitation at T0, T15 and T30. The lower left panel shows the corresponding microvascular resistances. The upper right panel shows the absolute coronary flow modification at baseline and after cardiac arrest resuscitation. The lower left panel shows the corresponding microvascular resistances

Baseline overall flow was 0.040 ± 0.03 L/min. Just after resuscitation (T0), a hyperaemic phase with significantly increased CBF (0.090 ± 0.06 L/min; *P* = 0.01) was observed. Mean aortic pressure significantly increased from 75 ± 10 mmHg at baseline to 153 ± 15 mmHg at T0 (*P* = 0.0002). Heart rate was significantly higher, with 94 ± 15 beats per minute (BPM) at baseline vs. 157 ± 48 BPM at T0 (*P* = 0.001). At T15 and T30, CBF significantly decreased respectively (0.026 ± 0.02 vs. 0.059 ± 0.05 L/min; *P* = 0.04). Mean aortic blood pressure remained stable and similar compared to baseline at T15 and T30 (75 ± 10 mmHg at baseline, 80 ± 17 mmHg at T15 and 76 ± 16 mmHg at T30). Heart rate remained stable at T15 and T30 and similar to baseline (94 ± 20, 101 ± 13 and 102 ± 13 beat per minute, respectively). Compared to baseline, 30 min after resuscitation, CBF was significantly lower with a 35% flow reduction (0.040 ± 0.03 vs. 0.026 ± 0.02 L/min; *P* = 0.03) (Fig. 3 and Table 1).

### Cerebral resistances

At baseline, cerebral resistances were 2014 ± 1015 WU. At T0 and T15, mean cerebral resistances remained stable and similar to baseline (1991 ± 1191 and 1967 ± 1048 WU, respectively). Mean microvascular cerebral resistances were significantly increased at T30 compared to baseline (3202 ± 1838 vs. 2014 ± 1015 WU; *P* = 0.04), (Fig. 3 and Table 1).

### Subgroup analysis according to no flow duration

No significant difference was observed in terms of flow and resistances between the group 1 and the group 2 at baseline, T0, T15 and T30 (*p* > 0.05 for all). We observed a similar hyperaemic phase at T0, with flow reduction and stabilisation at T15 and T30. Compared to T0, cerebral flow was lower at T15 in group 1 (0.096 ± 0.09 vs. 0.022 ± 0.01 L/min) and remained stable at T30 (0.028 ± 0.02 L/min). On the other hand, compared to T0, cerebral flow remained high at T15 in the group 2 (0.083 ± 0.01 vs. 0.095 ± 0.06 L/min) and decreased at T30 (0.025 ± 0.01 L/min). In the group 2, compared to baseline, we observed, higher microvascular resistances at T30 after resuscitation (2278 ± 1561 vs. 3658 ± 2218 WU) without reaching significant statistical difference (*P* = 0.11) (Table 1).

### Absolute coronary flow and resistances

We performed ABF and microvascular resistance measurements in seven left anterior descending and three right coronary arteries at baseline and at T30 in ten pigs. Compared to baseline, mean ABF was similar 30 min after cardiac arrest (0.143 ± 0.06 and 0.122 ± 0.05 L/min; *P* = 0.41). Mean coronary microvascular resistances were similar at baseline and at T30 (478 ± 181 and 563 ± 203 WU; *P* = 0.36). No statistical difference was observed between groups 1 and 2 (Table 2).

**Table 2:** Absolute coronary flow and microvascular resistances at baseline and after cardiac arrest resuscitation

	n vessels	Baseline	Post	*P* value
Flow (L/min)				
overall	10	0.122 ± 0.05	0.143 ± 0.06	0.41
3	5	0.140 ± 0.06	0.135 ± 0.06	0.99
5	5	0.117 ± 0.06	0.156 ± 0.07	0.55
Resistances (mmHg·min/l or WU)				
overall	10	563 ± 203	478 ± 181	0.36
3	5	508 ± 187	520 ± 223	0.99
5	5	569 ± 266	370 ± 108	0.40

## Discussion

In the present study, we show that this novel approach based on the continuous thermodilution principle allows simultaneous measurements of flow and microvascular resistances and monitor cerebral perfusion parameters after resuscitation from cardiac arrest. This approach has a number of potential advantages. First, this technique is simple, objective, accurate and reproducible [9, 12, 13]. Second, intravascular access simplifies experimental protocols, keeps the animals’ temperature stable and allows repeated measurements over time. Third, in this experimental model, the animal is its own control, with absolute measurements of flow, pressure, resistances and heart rate, which helps our understanding and interpretation of results.

OHCA remains a leading cause of death and permanent disability worldwide. Although patients are successfully resuscitated, they often suffer from internal organ injury, including hypoxic brain damage. Cardiac arrest initiates a complex cellular injury cascade, which compromises autoregulation and function of internal organs with a destructive systemic inflammatory response. The complexity of this cascade challenges the development of experimental models and effective treatment for cardiac arrest. Many experimental animal preparations have been developed to explore mechanisms of damage to vital internal organs after resuscitation from cardiac arrest and to develop targeted treatments. Porcine models of cardiac arrest and resuscitation offer several important advantages over other species, and outcomes in this large animal are readily translatable to the clinical setting [14]. We used a percutaneous intravascular measurement technique already available in clinical practice to accurately measure absolute flow and microvascular resistances in cerebral and coronary arteries based on thermodilution technique [9, 13]. Our study showed that 30 min after cardiac arrest and resuscitation, mean cerebral flow significantly decreased by 35%, and mean microvascular cerebral resistances significantly increased by 37% compared to baseline, while coronary flow and resistances remained stable, as did haemodynamic parameters (mean aortic blood pressure, heart rate and temperature). In agreement with literature data, we observed a hyperaemic phase just after resuscitation, with a significant increase in aortic blood pressure, heart rate and cerebral flow [8]. Our measurements add microvascular resistance evaluation, which is constant during this phase. It has been described that the absolute coronary flow technique induces maximal hyperaemia as compared with hyperaemia under adenosine in the coronary circulation [10]. Mechanism of brain injury induced by cardiac arrest remains unclear. According to our results, the hyperaemic phase just after resuscitation is probably due to haemodynamic modifications with constantly adaptive resistances, which means full cerebral circulation vasodilation from baseline to T15 and an increase in blood pressure and heart rate that increases cerebral flow. At a later phase (T30), we observed a significant increase in cerebral microvascular resistances with a decrease in cerebral flow. We were not able to show a significant difference between the two groups according to no flow duration (Additional files 1 and 2: Figures S1 and S2), probably due to the small sample size in each group and the considerable resuscitation failure in the 5-min group. Overall, our study showed that it is feasible to study cerebral flow and to assess microvascular resistances after cardiac arrest resuscitation in an animal setting to better understand complex haemodynamic modifications. This could allow to better explore therapies aiming to reduce cerebral microvascular resistances and to increase CBF in order to improve neurological outcome in OHCA patients.

### Limitations

As previously mentioned, the pig animal model has different cerebral vascular anatomy compared to humans. We performed this protocol in juvenile pigs, which are prone to have coronary and cerebral artery spasm during endoluminal instrumentation. Nevertheless, we administered intraluminal nitrates to prevent vasopasm and performed our measurements without significant haemodynamic modification. To rule out arterial spasm, we performed repeated angiograms. Furthermore, thermodilution technique could rule out arterial spasm within the arteria anatomica by the maintained ratio between the central arterial pressure and the distal pressure during flow measurements. We could not obtain a T30 measurement in one vessel due to cerebral vasospasm (Table 1). Pigs were anaesthetised and ventilated when inducing cardiac arrest; therefore, we had some differences in our model compared to OHCA in humans. However, the pig model remains an excellent large animal model of the human cardiovascular system [13]. In order to have reliable and accurate measurements, we kept the pressure wire in the same place at T0, T15 and T30; therefore, we were not able to measure contralateral cerebral artery and coronary circulation between T0 and T30. However, we did not observe any difference between the left and right cerebral arteries at T30 in our study which strengthens the interpretation of our results (Additional files 3 and 4: Figures S3 and S4). We have to acknowledge the small size of our subgroups, the limited evaluation period of 30 min after cardiac arrest and the difficulty to resuscitate pigs after 5 min of no flow. Lastly, in this feasibility study, we did not perform flow correlation with other techniques, including intracranial pressure measurement, downstream venous pressure measurement, brain imaging or cardiac output assessment during blood flow measurement. We also did not measure arterial PCO2, PO2 and haematocrit levels or other biological variables. These parameters could be, in purpose, of interest to explore this complex haemodynamic adaptation after CA resuscitation with this novel approach to assess and monitor brain perfusion in future studies.

## Conclusions

Cerebral blood flow and microvascular resistance measurement after cardiac arrest induction is feasible with thermodilution technique, allowing accurate monitoring and measurements of cerebral perfusion parameters. Our study showed significant early modifications of cerebral perfusion with blood flow decrease and increase of microvascular resistances after cardiac arrest resuscitation, while coronary circulation remained stable. This animal model simplifies evaluation of cerebral perfusion, a prerequisite to test innovative therapies to improve cerebral prognosis after cardiac arrest.

## Additional files

Additional file 1: **Figure S1.** Flow measurements in groups 3 min of No flow and 5 min of No Flow at baseline (pre) and after cardiac arrest resuscitation (post). (JPG 210 kb)

Additional file 2: **Figure S2.** Resistances measurements in groups 3 min of No flow and 5 min of No Flow at baseline (pre) and after cardiac arrest resuscitation (post). (JPG 254 kb)

Additional file 3: **Figure S3.** Flow measurements in groups left intracerebral artery (LIC) and right intracerebral artery (RIC) at baseline (pre) and after cardiac arrest resuscitation (post). (JPG 187 kb)

Additional file 4: **Figure S4.** Resistances measurements in groups left intracerebral artery (LIC) and right intracerebral artery (RIC) at baseline (pre) and after cardiac arrest resuscitation (post). (JPG 255 kb)

## Abbreviations

ABF: Absolute coronary blood flow
CA: Cardiac arrest
CBF: Cerebral blood flow
Fr: French
LIC: Left intracerebral artery
OHCA: Out of hospital cardiac arrest
*P*_d_: Distal pressure
*Q*: Flow
*Q*_i_: Infusion flow
*R*: Microvascular resistances
RIC: Right intracerebral artery
*T*: Temperature
*T*_i_: Infusion temperature
WU: Woods units
