# Supplementary figures and images for: A novel approach to assess cerebral and coronary perfusion after cardiac arrest
Source: Intensive Care Med Exp. 2018 Oct 12;6:39. doi: 10.1186/s40635-018-0204-3 (PMC6182017; doi:10.1186/s40635-018-0204-3)

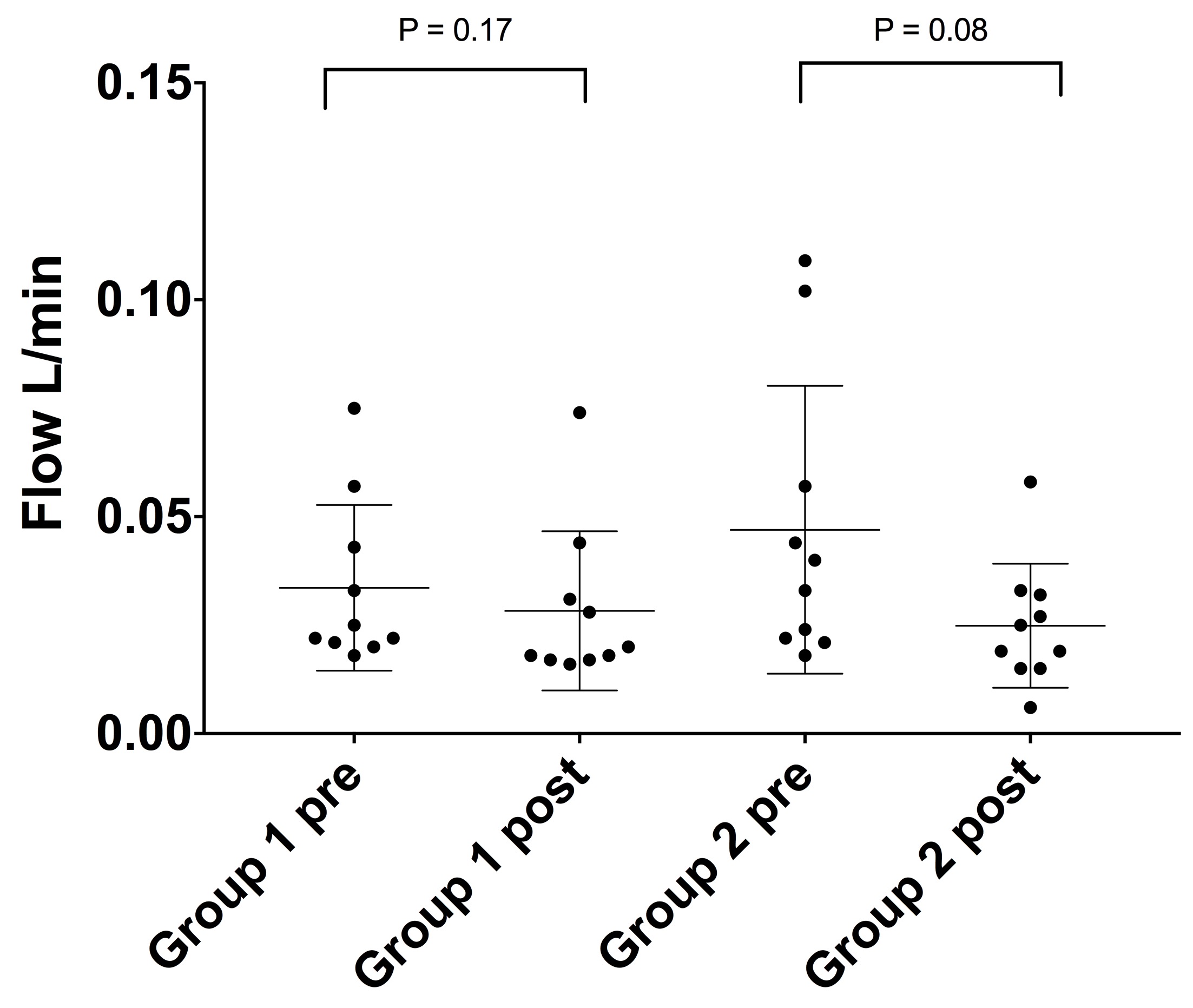

Supplement: Supplementary file 1 — Figure S1. Flow measurements in groups 3 min of No flow and 5 min of No Flow at baseline (pre) and after cardiac arrest resuscitation (post). (JPG 210 kb) [file 40635_2018_204_MOESM1_ESM.jpg]

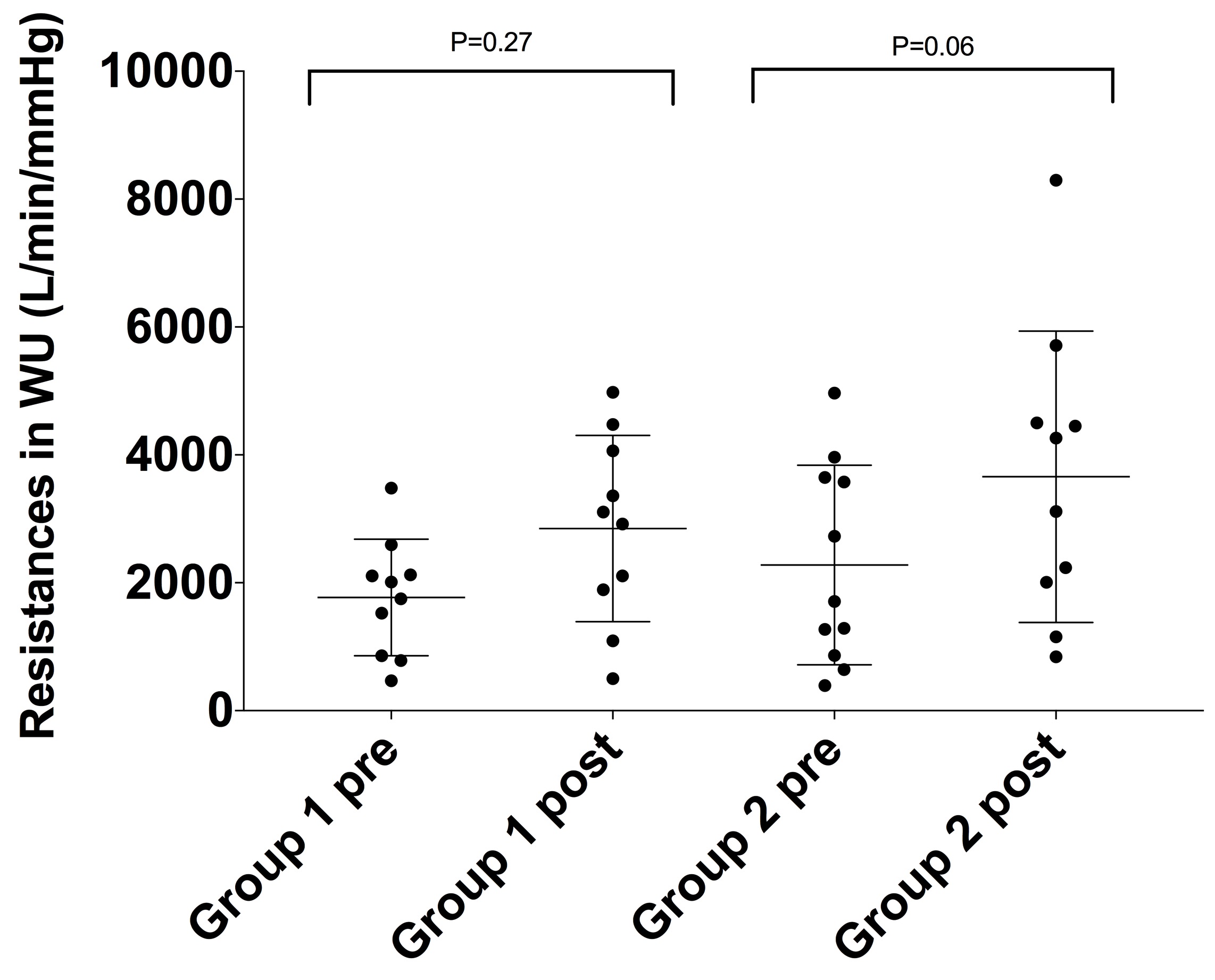

Supplement: Supplementary file 2 — Figure S2. Resistances measurements in groups 3 min of No flow and 5 min of No Flow at baseline (pre) and after cardiac arrest resuscitation (post). (JPG 254 kb) [file 40635_2018_204_MOESM2_ESM.jpg]

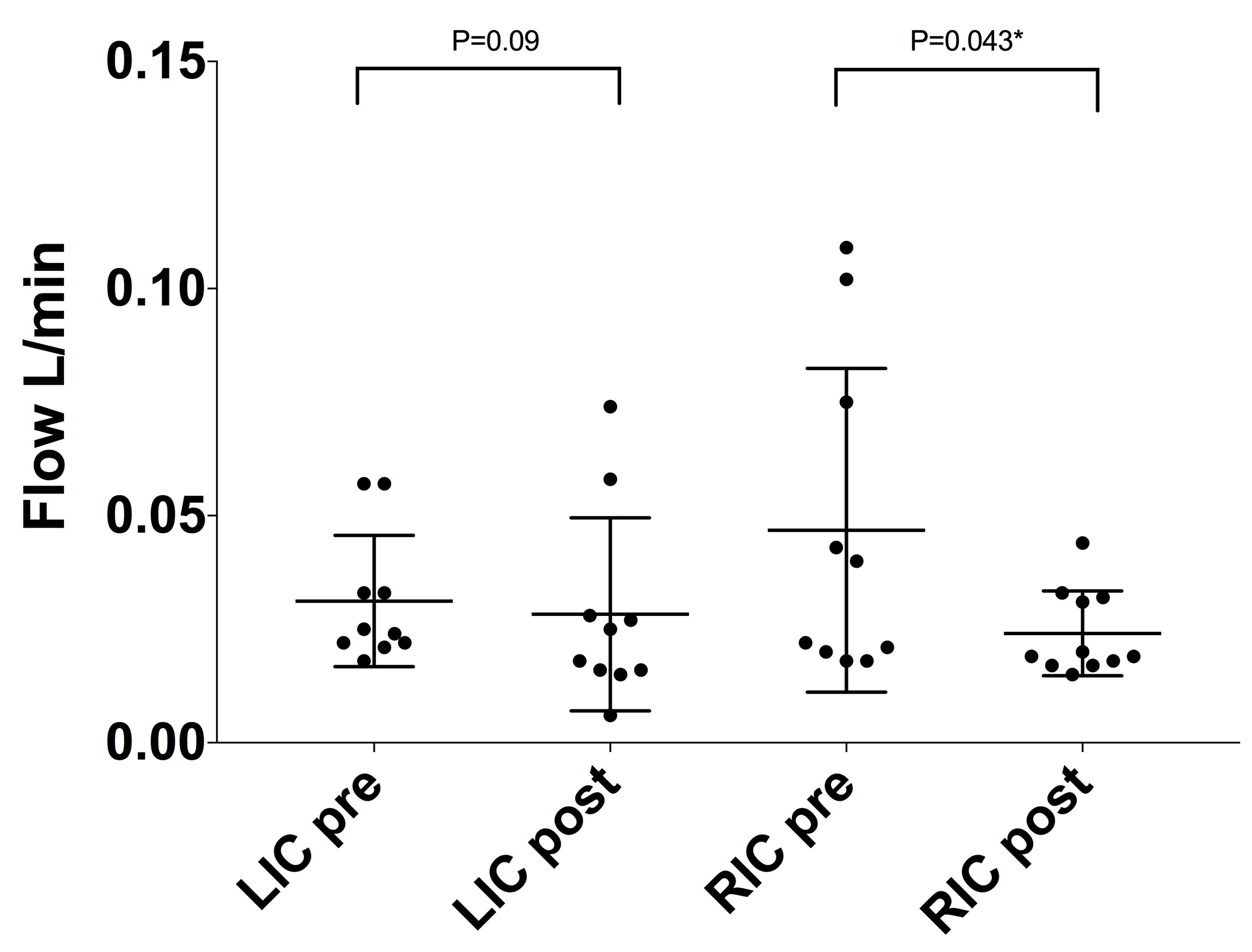

Supplement: Supplementary file 3 — Figure S3. Flow measurements in groups left intracerebral artery (LIC) and right intracerebral artery (RIC) at baseline (pre) and after cardiac arrest resuscitation (post). (JPG 187 kb) [file 40635_2018_204_MOESM3_ESM.jpg]

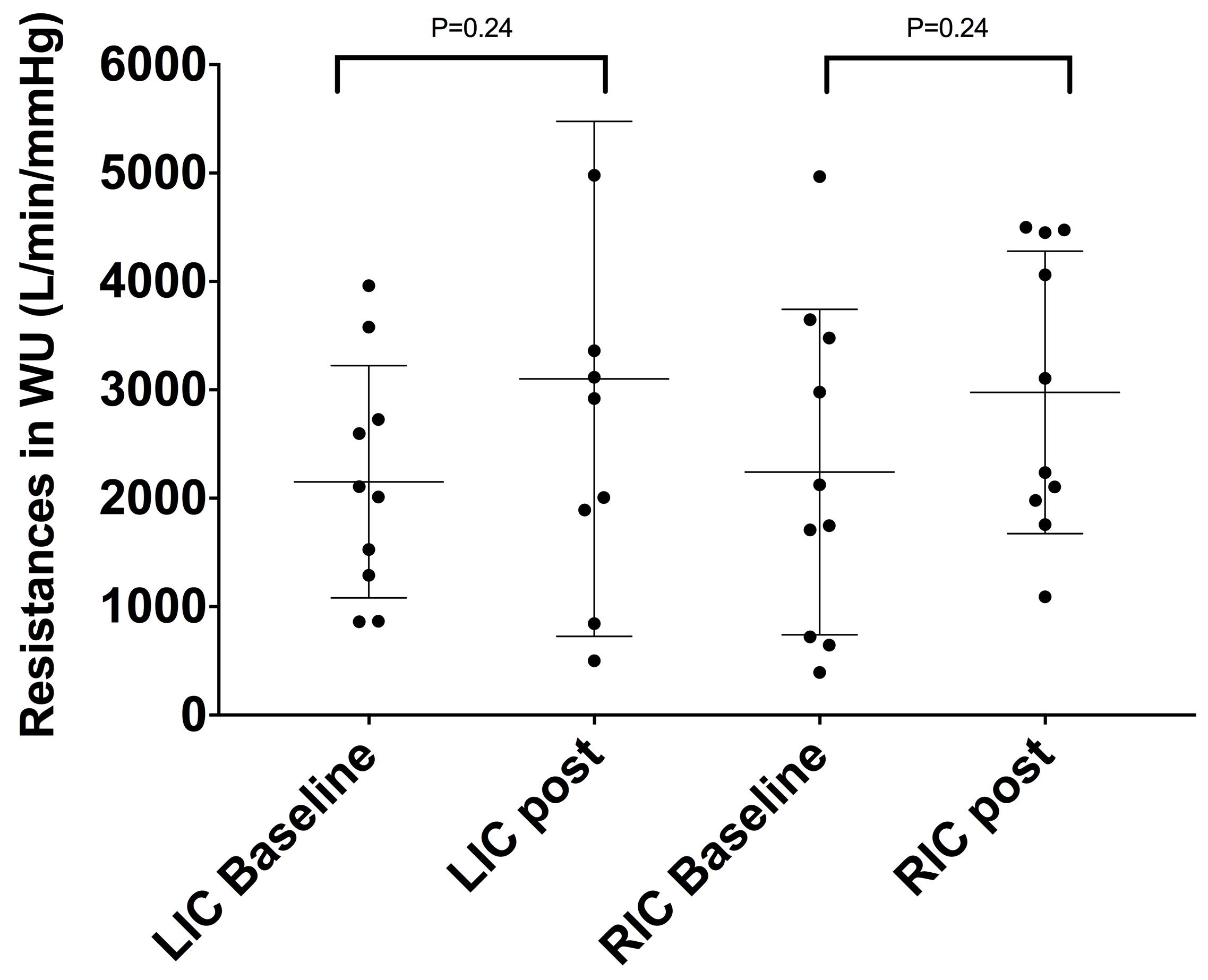

Supplement: Supplementary file 4 — Figure S4. Resistances measurements in groups left intracerebral artery (LIC) and right intracerebral artery (RIC) at baseline (pre) and after cardiac arrest resuscitation (post). (JPG 255 kb) [file 40635_2018_204_MOESM4_ESM.jpg]
